# A polygenic score for height identifies an unmeasured genetic predisposition among pediatric patients with idiopathic short stature

**DOI:** 10.1186/s13073-025-01455-3

**Published:** 2025-03-19

**Authors:** John P. Shelley, Mingjian Shi, Josh F. Peterson, Sara L. Van Driest, Jill H. Simmons, Jonathan D. Mosley

**Affiliations:** 1 Department of Biomedical Informatics, Vanderbilt University Medical Center, 1285 Medical Research Building IV, Nashville, TN 37232 USA; 2 Henry M. Jackson Foundation for the Advancement of Military Medicine, Washington, DC USA; 3 Uniformed Services University of the Health Sciences, Bethesda, MD USA; 4 Department of Medicine, Vanderbilt University Medical Center, Nashville, TN USA; 5 All of Us Research Program, National Institutes of Health (NIH), Bethesda, MD USA; 6 Department of Pediatrics, Vanderbilt University Medical Center, Nashville, TN USA

**Keywords:** Polygenic scores, Height, Common genetic variation, Short stature, Idiopathic short stature

## Abstract

**Background:**

A subset of children with short stature do not have an identified clinical explanation after extensive diagnostic evaluation. We hypothesized that a polygenic score for height (PGS_height_) could identify children with non-familial idiopathic short stature (ISS-NF) who carry a polygenic predisposition to shorter height that is not accounted for by existing measures.

**Methods:**

We studied 534 pediatric participants in an electronic health record (EHR)-linked DNA biobank (BioVU) who had been evaluated for short stature by an endocrinologist. Participants were classified as having one of five short stature subtypes: primary growth disorders, secondary growth disorders, idiopathic short stature (ISS), which was sub-classified into familial (ISS-F) and non-familial (ISS-NF), and constitutional delay of puberty (ISS-DP). Differences in polygenic predisposition between subtypes were analyzed using a validated PGS_height_ which was standardized to a standard deviation score (SDS). Adult height predictions were generated using the PGS_height_ and mid-parental height (MPH). Within-child differences in height predictions were compared across subtypes. Logistic regression models and AUC analyses were used to test the ability of the PGS_height_ to differentiate ISS-NF from growth disorders. The incremental improvement (ΔAUC) of adding the PGS_height_ to prediction models with MPH was also estimated.

**Results:**

Among the 534 participants, 29.0% had secondary growth disorders, 24.9% had ISS-F, 20.2% had ISS-NF, 17.2% had ISS-DP, and 8.6% had primary growth disorders. Participants with ISS-NF had similar PGS_height_ values to those with ISS-F (difference [Δ] in PGS_height_ SDS [95% CI] = 0.19 [− 0.31 to 0.70], *p* = 0.75). Predicted heights generated by the PGS_height_ were lower than the MPH estimate for children with ISS-NF (Δ[PGS_height_ − MPH] =  − 0.37 SDS; *p* = 3.2 × 10^−9^) but not for children with ISS-F (Δ =  − 0.07; *p* = 0.56). Children with ISS-NF also had lower PGS_height_ than children with primary growth disorders (ΔPGS_height_ =  − 0.53 [− 1.03 to − 0.04], *p* = 0.03) and secondary growth disorders (Δ =  − 0.45 [− 0.80 to − 0.10], *p* = 0.005). The PGS_height_ improved model discrimination between ISS-NF and children with primary (ΔAUC, + 0.07 [95% CI, 0.02 to 0.17]) and secondary growth disorders (ΔAUC, + 0.03 [95% CI, 0.01 to 0.10]).

**Conclusions:**

Some children with ISS-NF have an unrecognized polygenic predisposition to shorter height, similar to children with ISS-F and greater than those with growth disorders. A PGS_height_ could aid clinicians in identifying children with a benign, polygenic predisposition to shorter height.

**Supplementary Information:**

The online version contains supplementary material available at 10.1186/s13073-025-01455-3.

## Background

There has been considerable interest in translating polygenic scores for use in clinical settings to guide care decisions [1]. To date, these applications have largely focused on polygenic risk scores developed for the goal of identifying individuals at risk for adult-onset diseases, such as cancers or heart diseases [2]. There have been few clinical applications of these scores in pediatric patients, partly because many polygenic influences on clinical outcomes are age-dependent and scores are calibrated to predict these influences in adulthood [3–6]. Here, we describe an application of a polygenic score for adult height (PGS_height_) for identifying the etiology of pediatric short stature.

Pediatric short stature is a heterogeneous condition with causes ranging from benign genetic predispositions to occult diseases such as growth hormone deficiency or inflammatory bowel disease [7, 8]. Among patients referred to endocrinology for short stature, a subset of patients is found to have heights shorter than expected based on their family history in the absence of an identified growth disorder or other explanation. These children are classified as having idiopathic short stature of non-familial origin (ISS-NF). This diagnostic label can lead to extended periods of surveillance and testing and resultant parental and child anxieties surrounding a potentially undiagnosed underlying disease [9, 10]. Recent studies have shown that chromosomal microarray and genetic sequencing can identify a monogenic etiology in up to 27% of patients with ISS-NF [11]. Indeed, the American College of Medical Genetics has recently recommended chromosomal microarray as part of the initial evaluation of ISS, but sequencing is only recommended in patients that have clinical features (e.g., disproportionate short stature) or a family history (e.g., parent with short stature) that is concerning for a monogenic form of short stature [12]. This approach is imprecise, however, as many of the most common monogenic causes of ISS, such as SHOX deficiency, have highly variable and often subtle clinical presentations [13].

Another possible genetic explanation for ISS-NF is a predisposition to shorter stature that is not captured by traditional assessments like the mid-parental height (MPH) or the family history. Some children may receive a skewed distribution of height-associated genetic variants from their parents which could lead to inaccurate height prediction. Supporting this hypothesis, studies of children and adults in population-based cohorts have shown that the combination parental heights and polygenic scores for height improves the accuracy of height predictions, as compared to parental heights alone [14, 15].

We hypothesized that a PGS_height_ could aid in identifying children with ISS-NF who have a polygenic predisposition to shorter height that is not captured by parental heights and distinguish them from children with growth disorders. We first validated the association between the PGS_height_ and short stature using a pediatric cohort. We then tested whether children evaluated for unexplained short stature have a benign genetic predisposition to short stature and whether the PGS_height_ could improve discrimination between ISS-NF and growth disorders when compared to mid-parental height (MPH).

## Methods

### Study populations

All data for participants were obtained from BioVU, a DNA biobank linked to a deidentified copy of the electronic health record (EHR) [16]. BioVU comprises DNA extracted from discarded blood samples from approximately 325,000 participants receiving healthcare at Vanderbilt University Medical Center (VUMC). Participants were consented prior to enrollment in BioVU with parental consent obtained for pediatric participants. This study was evaluated by the Vanderbilt Institutional Review Board (IRB #180267) and determined to be non-human subjects research as data are fully deidentified [17].

#### Overall BioVU cohorts

To validate the PGS_height_, we identified a cohort of adult and pediatric participants with genotyping in BioVU who did not have a diagnosis of a disease that can lead to short stature. A total of 70,467 genotyped participants of European ancestry were identified and participants with an ICD code for a disease associated with short stature were excluded (Additional file 1: Table S1). For the adult cohort, participants were excluded if they had fewer than 2 height measurements between the ages of 20 and 70 years. For the pediatric cohort, participants were excluded if they had fewer than 2 height measurements between the ages of 2 and 19 years. The final cohorts included 33,637 adult and 5581 pediatric participants.

#### Short stature cohort

The study cohort was derived from a set of 758 children who were evaluated by a pediatric endocrinologist on the same day that an ICD-9 or ICD-10 code for short stature appeared in their clinical record. ICD codes used to select this cohort are listed in Additional file 1: Table S2. Participants were excluded prior to chart review if they had a known genetic syndrome, were born severely preterm (less than 32 weeks), or had prior treatment with growth hormone. After chart review, participants were excluded if the short stature diagnosis was inconclusive (more than one short stature etiology identified or lost to follow-up) or if they were missing initial visit or parental heights.

### Clinical data

For each participant, all endocrinology notes were manually reviewed by a medical student (JPS) and a clinician (JDM) in consultation with a pediatric endocrinologist (JHS). The “initial visit height” was the height measured on the same day as the initial endocrinology visit. If not available on the same day, the height measurement occurring within 1 year and closest to the initial visit date was selected. The R package childsds was used to convert initial visit height measurements to age- and sex-specific standard deviation scores using the Centers for Disease Control and Prevention’s (CDC) growth reference standards [18, 19].

Parental heights were extracted through chart reviews. These heights were converted to a sex-specific SDS using the National Health and Nutrition Examination Survey (NHANES) cohorts as the reference standard for the adult population in the USA [20]. For the SDS, 0 was defined as the mean height among NHANES adults of each participant’s sex and the standard deviation was defined as the standard deviation of the distribution in these adults. The NHANES data is further described in Additional file 2. The mid-parental height (MPH) for a child was calculated as 0.72 × (average of mother and father’s height SDS values), as recommended in guidelines from the Pediatric Endocrine Society [21, 22].

The etiology of short stature determined by the evaluating endocrinologist was extracted through chart review of all clinical notes. The final diagnosis was classified as one of the following: familial ISS (ISS-F), non-familial ISS (ISS-NF), ISS due to constitutional delay of puberty (ISS-DP), primary growth disorders, and secondary growth disorders. These categories follow the 2015 International Classification of Pediatric Endocrine Diagnoses (ICPED) guidelines [23]. Participants were classified as ISS-F if they had an initial visit height within the expected target range (± 1.6 s.d.) according to the MPH. Primary growth disorders included clinically defined syndromes associated with short stature and small for gestational age without catch-up growth (SGA). Secondary growth disorders included growth hormone deficiency, non-endocrine systemic diseases, insufficient nutrient intake, and iatrogenic short stature due to systemic glucocorticoid use. Children with short stature due to constitutional delay of puberty were classified as ISS-DP. Children with short stature of unknown etiology after endocrinology evaluation and follow-up were classified as ISS-NF. Children originally diagnosed with growth hormone deficiency but with normal growth hormone results (maximum growth hormone response ≥ 10 ng/ml) were re-categorized as ISS-NF.

### Genetic data

Single-nucleotide polymorphism (SNP)-based genotyping was performed using the Illumina MEGA-Ex platform. SNPs were imputed to the Haplotype Reference Consortium (HRC) release 1.1 haplotypes using the Michigan Imputation Server [24]. Quality control was performed using PLINK 2.0 [25]. Closely related individuals were excluded by randomly removing one of each pair of individuals with a kinship coefficient (pi-hat) greater than 0.2. Participants with genotype missingness greater than 2% were excluded as were participants with outlying heterozygosity defined as a heterozygosity more than 4 standard deviations from the mean. Genetic ancestry was determined by principal components (PCs) analysis in conjunction with HapMap reference populations [26]. Within-ancestry PCs to adjust for residual population stratification were calculated using the SNPRelate R package [27].

### Development of the PGS

These analyses utilized a previously validated PGS for adult height (PGS_height_) which was derived from a GWAS of over five million adults (76.8% European) across 281 studies [15]. In that study, SNP effect estimates for the PGS study were generated using SBayesC [28]. Weights specific to the European ancestry subset were downloaded from the GIANT Consortium website [29]. Of the 1,098,854 SNPs with available weights, 1,096,598 (99.8%) were available in our imputed dataset (imputation *R*^2^ ≥ 0.7). The PGS_height_ was computed for each child by summing the product of each allele weight and the allele dosage across all SNPs. The PGS_height_ was then converted to an SDS, defining 0 as the mean PGS_height_ SDS among the cohort of BioVU adults without an ICD code for a potentially pathologic cause of short stature as described earlier. The standard deviation for the SDS was defined as the standard deviation of the PGS_height_ distribution in these adults. The pediatric population was not used as the reference for the SDS to avoid selection bias due to an enrichment of patients undergoing evaluation for short stature in our biobank.

### Statistical analyses

Baseline characteristics of the cohorts were summarized as counts (frequencies) for binary variables and median (interquartile ranges [IQR]) for continuous variables.

In the adult BioVU cohort, multivariable linear regression models were used to test the association between a person’s PGS_height_ and the median value of all available height measurements for an individual, adjusting for the top 10 principal components (PCs). In the pediatric cohort, multivariable logistic regression models, also adjusting for the top 10 PCs, were used to test the association between the PGS_height_ and meeting the threshold for short stature, defined as a height ≥ 2 standard deviations below the mean.

In the short stature cohort, one-sample Wilcoxon signed rank tests were used to determine whether the median MPH and PGS_height_ were shorter than expected based on adult population averages (i.e., whether either SDS differed from 0). Tukey’s honest significant difference (HSD) test was used to estimate and test the significance of pairwise differences in the PGS_height_ SDS between short stature subtypes. To enable direct comparisons of predicted height estimates based on the PGS_height_ and MPH, the PGS_height_ SDS was converted to a predicted adult height using a model developed in the adult BioVU cohort (see Additional file 2 for full details). Paired-sample Wilcoxon signed rank tests were utilized to test the significance of the within-person difference between the MPH-based and PGS_height_-based adult height predictions by diagnostic subgroup. In participants with a primary or secondary growth disorder, we tested the significance of the within-person height prediction difference stratifying by the growth disorder subtype.

In participants with ISS (any subtype), we tested whether the PGS_height_ differed by monogenic risk for short stature which we defined as having a parent with short stature (height SDS <  − 2). We then compared the PGS-based height predictions between the two risk strata and tested the significance of the within-person prediction difference. This analysis was repeated in the subset of participants with an initial visit height SDS <  − 2.

In participants with measured heights in adulthood (*N* = 122), we calculated the difference between adult height and the MPH- and PGS-based height predictions. Adult height was defined as the median if there was more than 1 height measurement for a participant. We first compared MPH- and PGS-based height predictions and then compared the discrepancy between MPH- and PGS-based predicted and measured adult height. In the subset of participants with ISS (*N* = 45), we calculated the variance in adult height explained (*R*^2^) by the MPH, PGS_height_, and linear combination of the MPH and PGS_height_ using simple linear regression. Due to the limited sample size in this subset, we estimated the *R*^2^ distribution using bootstrapping with 5000 replicates.

We then tested whether the PGS_height_ could improve clinical discrimination between patients with ISS-NF and patients with primary or secondary growth disorders. To test the association of the PGS_height_ with receiving an ISS diagnosis, two multivariable logistic regression models were fit: (1) a baseline model that included a child’s sex, age at the initial visit, and the top 10 within-ancestry PCs and (2) the baseline model with the addition of the PGS_height_. This PGS_height_ model was used to generate predicted probabilities of ISS-NF (versus growth disorders) for each participant. Area under the curves (AUC) were then derived from these models and their 95% confidence intervals were estimated using bootstrapping with 5000 replicates. The improvement in discrimination with addition of the PGS_height_ was assessed by calculating the change in AUC (reported as ΔAUC), estimating 95% confidence intervals using bootstrapping with 5000 replicates. In a sensitivity analysis, the baseline model was additionally adjusted for initial visit height. Additional sensitivity analyses examined subgroups of children: (1) falling outside of MPH-based genetic height predictions (discrepancy between child’s measured height SDS and MPH SDS ≥ 2 s.d.) and (2) meeting the clinical definition of short stature at the initial visit (height SDS ≤  − 2) [22].

We repeated this discrimination analysis to test a single variable approach to integrating the PGS_height_ into the short stature evaluation. We calculated the difference between the initial visit height and the PGS-based height prediction. This is the approach currently used by clinicians to identify children deviating from their MPH-based height predictions [21]. We also tested the discrimination of this traditional, MPH-based measure and an average of the PGS- and MPH-based measures. In addition to the AUC, the optimal discrimination threshold, sensitivity, specificity, PPV, and NPV were estimated using bootstrapping with 5000 replicates. The optimal discrimination threshold was determined using the Youden index [30]. These analyses utilized the R package pROC for ROC curve analysis, the packages boot and boot.pval for bootstrapping, and the package cutpointr for calculating the optimal threshold and ROC curve performance metrics [31–33].

In association analyses, statistical tests were two-sided and a *p* value < 0.05 was considered significant. In discrimination analyses, AUC confidence intervals were estimated using the bias-corrected and accelerated (BCa) bootstrap. A change in AUC with a *p* value < 0.05 was considered significant. All analyses were performed using R version 4.0.2.

## Results

### Lower PGS_height_ associates with pediatric short stature

**Fig. 1:**
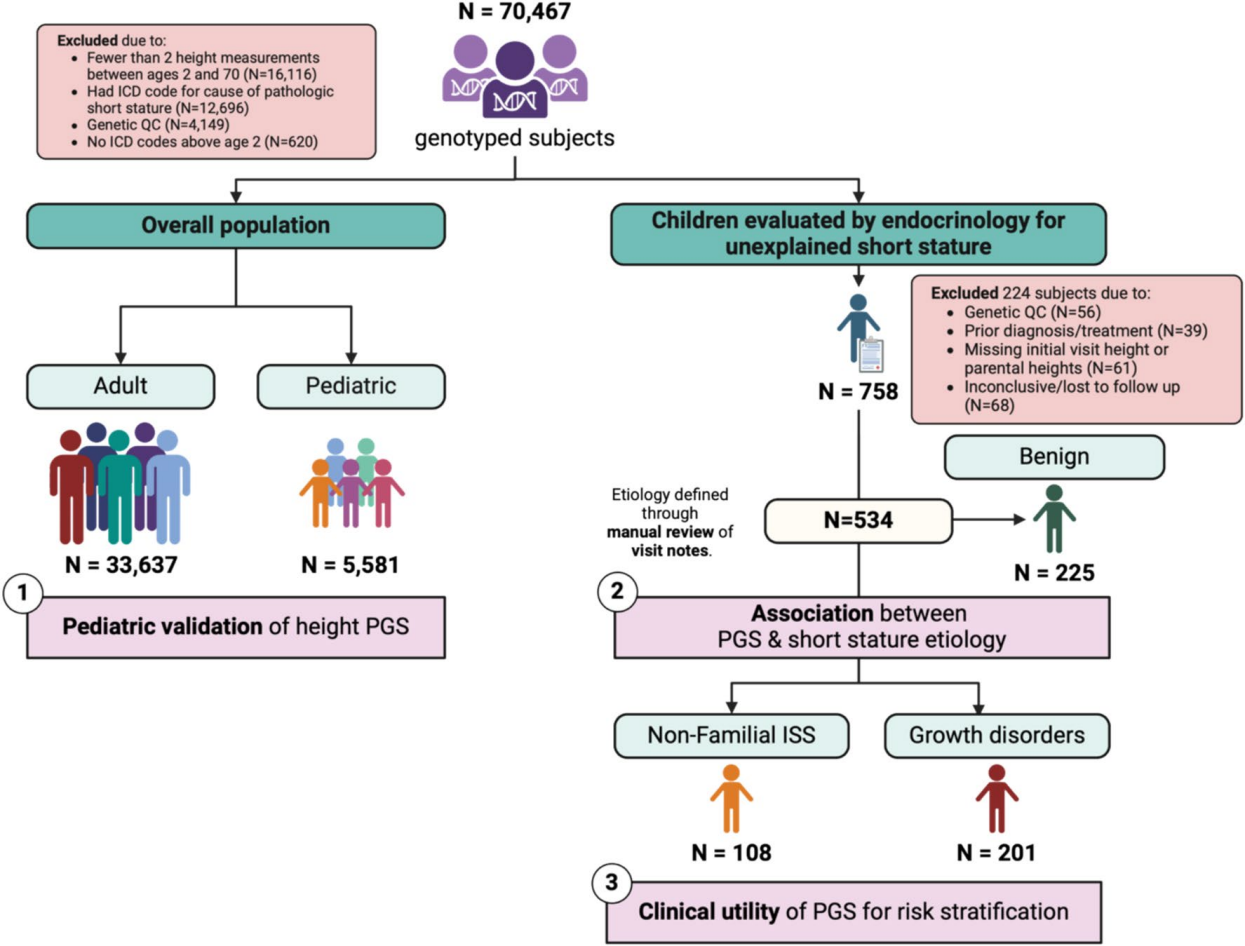
Outline of study populations and aims. “Benign” refers to the causes of idiopathic short stature (ISS) that have a defined cause: familial ISS (ISS-F) or constitutional delay of puberty (ISS-DP)

**Fig. 2:**
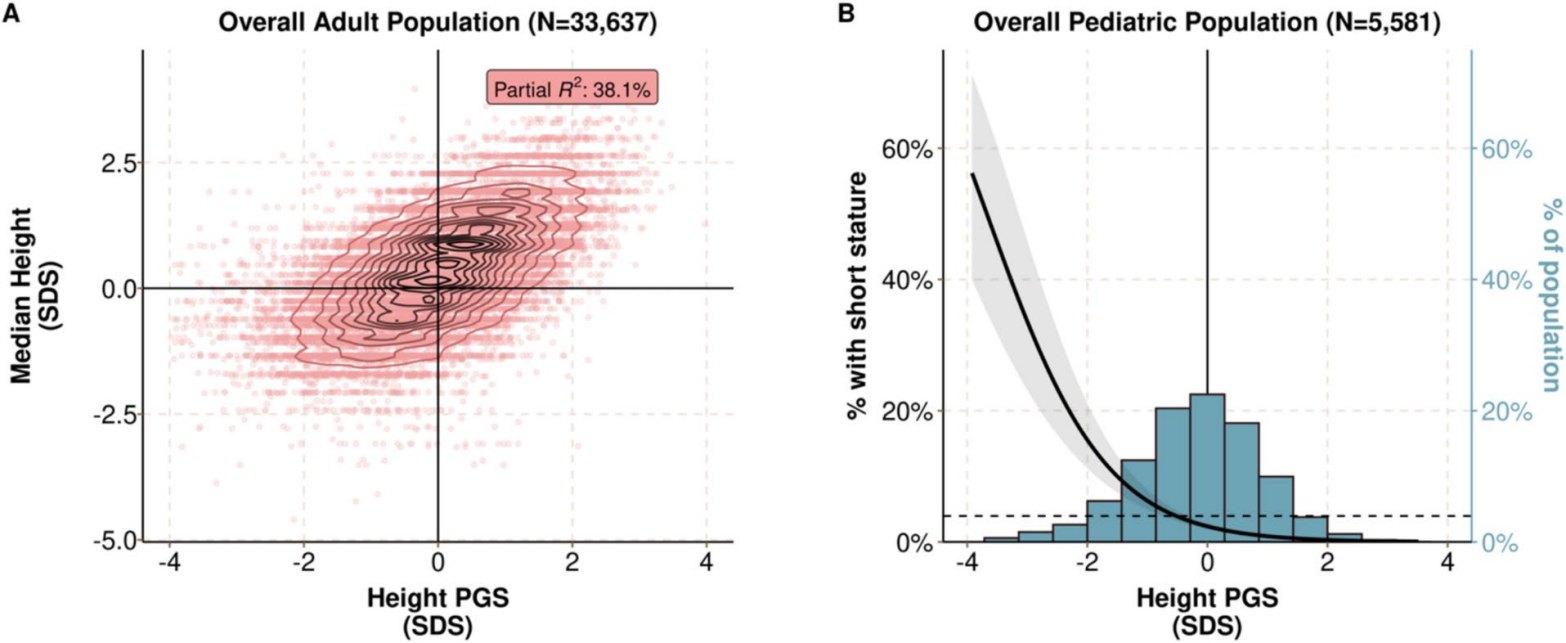
A polygenic predisposition to height (PGS_height_) is associated with adult height and prevalent short stature in BioVU. **A** Scatter plot comparing the PGS_height_ to age- and sex-normalized heights among adults (*n* = 33,637). Height was defined as the median of all of a given subject’s height measurements. The partial *R*^2^ is from a linear model adjusting for the top 10 within-ancestry principal components. **B** Histogram of the PGS_height_ distribution in the pediatric population and the association between the PGS_height_ and clinical short stature (median height 2 or more standard deviations below the reference standard) (*n* = 221 [4.0% of 5581]). The dashed line reflects the prevalence of short stature in the population and the left y-axis reflects the predicted probability of short stature with a lower PGS_height_. The right y-axis reflects the percent of the pediatric population with a given PGS_height_

There were 33,637 adult participants and 5581 pediatric participants selected for analysis (Fig. 1). Descriptive statistics are listed in Additional file 1: Table S3. Most participants were female (60.0% in the adult population, 57.1% in the pediatric population). The mean age was 49.6 years (SD 15.0) for adults and 13.3 years (SD 6.3) for children. Among the adult population, there was a median of 10 (interquartile range [IQR]: 5 to 22) height measurements per person over 11.3 (5 to 17.2) years. Among the pediatric population, there was a median of 7 (IQR: 3 to 16) height measurements per person over 10.2 (5.5 to 15.5) years. The PGS_height_ explained 38.1% of height variation among adults (Fig. 2A). Among children, the PGS_height_ was inversely associated with meeting the criteria for short stature, defined as a median height more than 2 standard deviations below the expected mean for the patient’s age and sex (odds ratio (OR): 0.36 (0.31–0.43); *p* = 2 × 10^−35^) (Fig. 2B).

In sum, the PGS_height_ explained a significant portion of height variance in adults, and a polygenic predisposition to shorter height was associated with an increased likelihood of meeting the diagnostic criteria for short stature among children.

### Children with ISS have a lower PGS_height_ than other subgroups

There were 534 participants who underwent an evaluation in pediatric endocrinology clinic for unexplained short stature (Fig. 1). The median follow-up time was 3 (IQR, 2 to 9) visits over a median 1.9 (0.3 to 4.7) years. Within this cohort, 333 (62.4%) children were diagnosed with ISS and 201 (37.6%) were diagnosed with primary or secondary growth disorders. Among participants diagnosed with ISS, the most common form was ISS-F which was defined as having an initial visit height within the expected range of the MPH (Table 1). Among participants diagnosed with primary or secondary growth disorders, the most common diagnoses were growth hormone deficiency (*N* = 76), non-endocrine systemic disease (*N* = 60), and syndromic short stature (*N* = 34). The non-endocrine systemic diseases most commonly attributed to short stature were inflammatory bowel disease (*N* = 14 of 60, 23.3%), celiac disease (*N* = 7, 11.7%), and cystic fibrosis (*N* = 5, 8.3%). The most common causes of syndromic short stature were neurofibromatosis type 1 (*N* = 4 of 34, 11.7%) and mosaic Turner syndrome (*N* = 2, 5.9%).

**Table 1:** Descriptive statistics for short stature cohort

Characteristic	Outcome
***N***	534
**Female, no. (%)**	28.3%
**Age (years) at initial visit**	
Median (IQR)	10.7 (7.5 to 13.2)
**Height SDS at initial visit**^**a**^	
Median (IQR)	−2.05 (−2.46 to −1.59)
Height SDS ≤ −2, no. (%)	285 (53.4%)
Deviation from MPH ≥ 1.6 s.d., no. (%)	401 (75.1%)
**Adult height SDS**^**b**^	
Available, no. (%)	122 (22.8%)
Median (IQR)	−0.92 (−1.73 to −0.42)
**Genetic height predictors, median (IQR)**	
Mid-parental height SDS^c^	0.03 (−0.26 to 0.38)
PGS_height_ SDS^d^ (z score)	−0.86 (−1.52 to −0.22)
**Length of follow-up**	
Number of visits, median (IQR)	3 (2 to 9)
Number of years, median (IQR)	1.9 (0.3 to 4.7)
**Testing, no. (%)**	
Bone age	494 (92.5%)
IGF-1	484 (90.6%)
Growth hormone stimulation	187 (35.0%)
**Initiated growth hormone treatment, no. (%)**	215 (40.3%)
**Etiology of short stature, no. (%)**	
*Idiopathic short stature*	
Familial (ISS-F)	133 (24.9%)
Non-familial (ISS-NF)	108 (20.2%)
Constitutional delay of puberty (ISS-DP)	92 (17.2%)
*Primary growth disorders*	
Syndromic short stature	34 (6.4%)
Small for gestational age, no catch-up growth	12 (2.2%)
*Secondary growth disorders*	
Growth hormone deficiency	76 (14.2%)
Non-endocrine systemic disease	60 (11.2%)
Insufficient nutrient intake	16 (3.0%)
Systemic glucocorticoid use	3 (0.6%)

^a^Height SDS was calculated by standardizing pediatric heights to CDC reference standards

^b^Adult height SDS was calculated as the median of all height measurements in any clinical setting at or above the age of 18

^c^Mid-parental height was standardized to the NHANES adult population as described in the Supplemental Material

^d^PGS_height_ SDS was calculated by standardizing to the adult BioVU dataset, specifying a mean of 0 and a standard deviation of 1

**Fig. 3:**
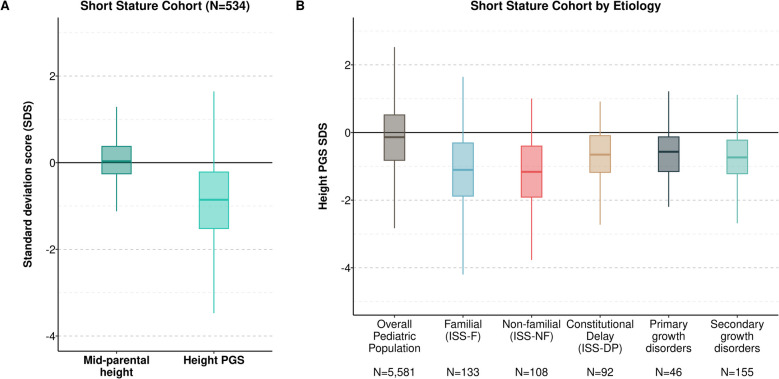
Children referred for short stature have a polygenic predisposition to shorter height, which is greatest for familial and non-familial ISS. **A** Box-and-whisker plot showing the PGS_height_ and the MPH SDS among participants in the short stature. The MPH SDS reflects the sex-adjusted standardized height relative to the NHANES reference standard, while the PGS_height_ SDS reflects the standardized PGS_height_ relative to the BioVU adult population. **B** Violin plot comparing PGS_height_ SDS by short stature etiology in the short stature cohort. The overall pediatric population is included as a reference

The median height SDS at the initial visit was − 2.05 (IQR, − 2.46 to − 1.59) and the median PGS_height_ SDS was − 0.86 (IQR, − 1.52 to − 0.22) (Fig. 3A). Compared to the general population of pediatric patients in BioVU, PGS_height_ was lower across all short stature subtypes. We first tested whether height and PGS_height_ differed between children with ISS who had an identified familial origin (ISS-F) and who did not (ISS-NF). Children diagnosed with ISS-NF were shorter on average than children with ISS-F (Δ =  − 0.90 s.d. [CI, − 1.12 to − 0.67], Tukey’s *p* = 3.0 × 10^−10^) but the PGS_height_ did not differ (− 0.004 [− 0.37 to 0.36], *p* = 0.99) (Fig. 3B, Additional file 2: Fig. S1). Children with ISS-NF were also shorter than children with primary growth disorders (− 0.70 [− 1.00 to − 0.40], *p* = 5.2 × 10^−9^) but had similar heights to children with secondary growth disorders (− 0.004 [− 0.22 to 0.21], *p* = 0.99). Children with ISS-NF had a lower PGS_height_ than children with primary (− 0.54 [− 1.03 to − 0.04], *p* = 0.03) and secondary growth disorders (− 0.45 [− 0.80 to − 0.10], *p* = 0.005). PGS_height_ differences between diagnostic subtypes were similar when restricting to children with an initial visit height SDS <  − 2 (Additional file 2: Fig. S2).

These results demonstrate that children with ISS-F and ISS-NF carry a similar polygenic predisposition to short stature. Children with secondary growth disorders and ISS-NF did not differ in stature at the initial visit, but children with ISS-NF carried a stronger predisposition to shorter stature.

### Adult height predictions based on MPH and PGS_height_ are most discordant in ISS-NF

**Fig. 4:**
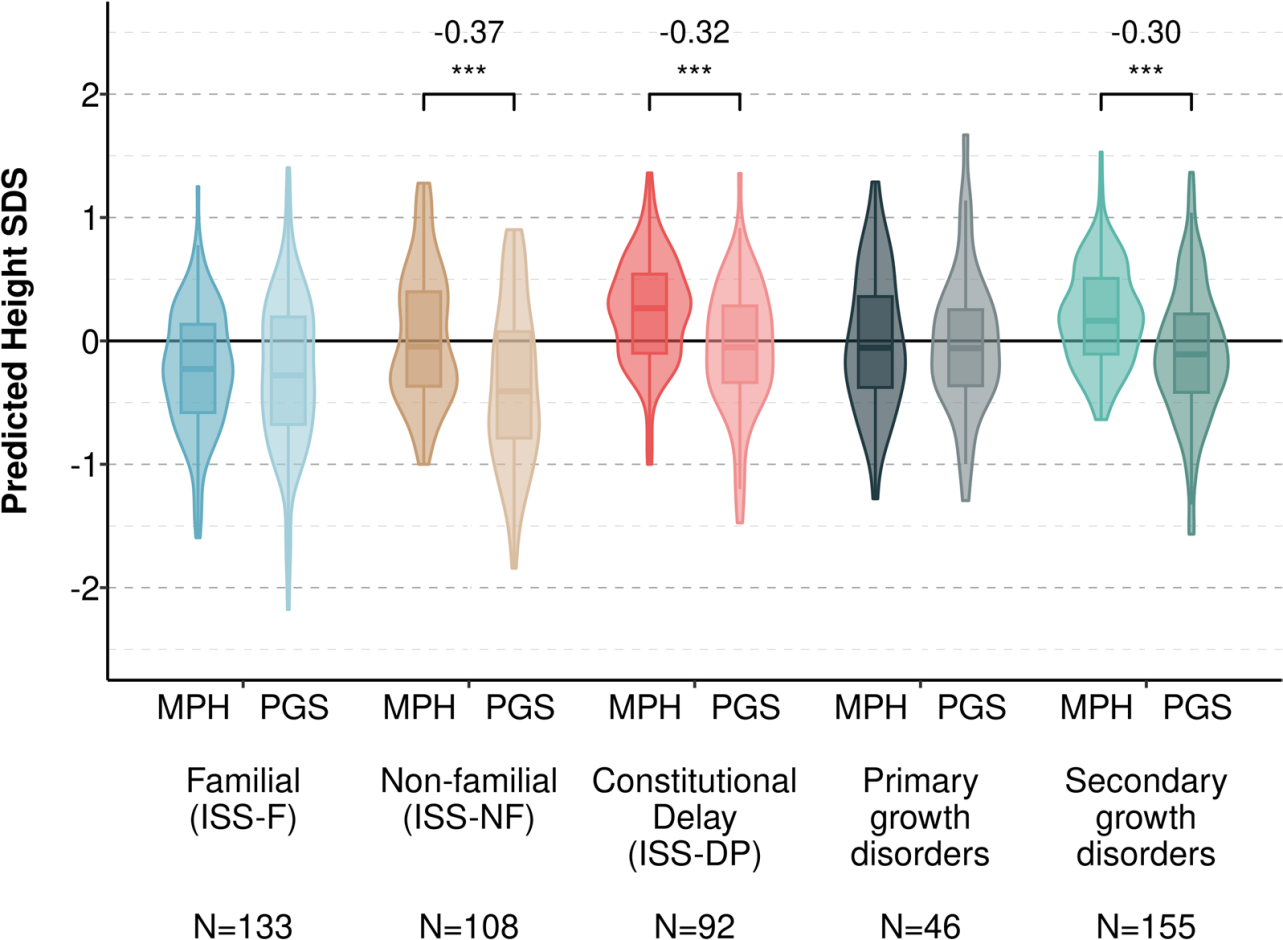
Predicted heights based on the PGS_height_ are lower, compared to MPH-based heights, except for children with familial ISS. Violin plot comparing height predictions by method (MPH and PGS_height_) for each subgroup based on short stature etiology. The PGS_height_ was converted to a predicted adult height by modeling height as a function of PGS_height_ and 10 principal components in BioVU adults. The median within-person difference in height predictions using the PGS_height_ and MPH is shown above each subgroup. Paired samples Wilcoxon signed rank tests were used to test the significance of within-person differences. ***: Wilcoxon *p* < 0.001

We then tested whether genetic height predicted using parental heights (i.e., MPH) were discordant from predictions using the PGS_height_. Discordance between the measures could indicate that the MPH is misestimating height potential and leading to misclassification in children with heights outside of the range of their MPH. Among children diagnosed with ISS-F and primary growth disorders, PGS-predicted adult height and MPH-predicted adult height were concordant (Fig. 4). However, PGS-predicted heights were lower than MPH among children with ISS-NF (Δ =  − 0.37; *d* =  − 0.64; *p* = 3.2 × 10^−9^), ISS-DP (Δ =  − 0.32; *d* =  − 0.61; *p* = 1.5 × 10^−7^), and secondary growth disorders (Δ =  − 0.30; *d* =  − 0.54; *p* = 1.4 × 10^−9^). In the subset with an initial visit height SDS <  − 2, results were similar except for ISS-F where the PGS-predicted adult height was higher than the PGS-predicted height (Δ = 0.43; *d* = 0.76; *p* = 0.005) (Additional file 2: Fig. S3). Within the pathologic growth disorder group, height predictions were lower when using PGS_height_ for children diagnosed with growth hormone deficiency and systemic disease but not children with syndromic short stature (Additional file 2: Fig. S4). This pattern of differences in height prediction methods was also true in the height SDS <  − 2 subset (Additional file 2: Fig. S5).

We then tested whether the PGS_height_ was comparable to MPH for the prediction of adult heights in a subset of 122 children with measured height data available in adulthood (age ≥ 18). For patients with ISS-NF, the difference between the measured and predicted adult height SDS was lower when using the PGS_height_ (Δ =  − 0.37 [IQR, − 1.08 to − 0.11]) compared to MPH (− 1.23 [− 1.66 to − 0.82]; Additional file 2: Fig. S6). The PGS_height_ explained 31% and 56% of the variance in adult heights in patients with ISS-F and ISS-NF, respectively (Additional file 2: Fig. S7). A linear combination of the PGS_height_ and MPH explained more variance than either predictor alone with a combined model explaining 54% and 62% of the variance in adult heights with ISS-F and ISS-NF.

Having shown that some participants carry a stronger polygenic predisposition than estimated using MPH, we tested whether the PGS- and MPH-based height predictions differed when stratifying by monogenic short stature risk. We restricted this analysis to participants diagnosed with either ISS-F or ISS-NF. In children with higher monogenic risk (*N* = 18), PGS-predicted height was higher than MPH (ΔSDS = 0.56; *d* = 0.47; *p* = 5.1 × 10^−16^) predictions (Additional file 2: Fig. S8). In the subset of high-risk children with height SDS <  − 2, PGS-predicted height was also higher than MPH (ΔSDS = 0.91; *d* = 1.56; *p* = 1.0 × 10^−4^) predictions. We then compared PGS-predicted height between participants with low or high monogenic risk. PGS-predicted height was similar in children regardless of risk. However, in the height SDS <  − 2 subset, the PGS-predicted height was 0.43 s.d. higher in the children at higher monogenic risk (*d* = 0.58; *p* = 0.015) suggesting that these children have a weaker polygenic predisposition to short stature compared to the genetic predisposition captured by the MPH.

In summary, we demonstrate that the PGS_height_ and MPH are complementary predictors of adult height, and that children with ISS-NF carry a polygenic predisposition to short stature that is not captured by the MPH. In contrast, patients with ISS at higher risk for monogenic short stature had less of a polygenic predisposition than predicted by their MPH. Finally, integration of the two genetic height predictors improved prediction of final, adult heights in patients with ISS-F and ISS-NF.

### PGS_height_ improves model discrimination between ISS-NF and pathologic growth disorders

**Fig. 5:**
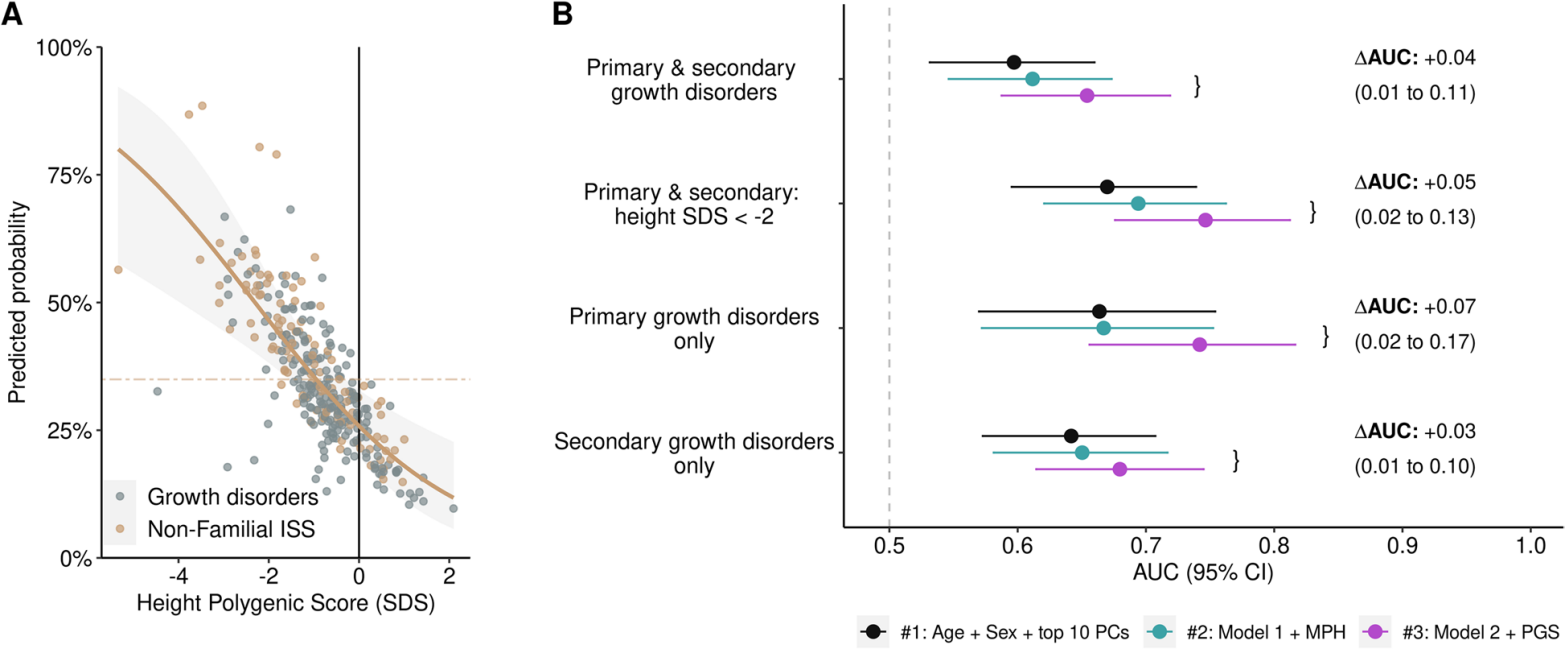
The PGS_height_ improves model discrimination between children with ISS-NF and pathologic growth disorders. **A** Scatter plot showing the relationship between the PGS_height_ and receiving a diagnosis of ISS-NF in the cohort of children with either ISS-NF or pathologic growth disorders. Logistic regression, adjusting for 10 PCs, was used to generate predicted probabilities of ISS. The gold line denotes the baseline probability of being classified as having ISS-NF. **B** Forest plot comparing discrimination (AUC) between ISS-NF and pathologic growth disorders when using a model with only age, sex, and 10 PCs (black), with addition of the MPH (blue) and with addition of the PGS_height_ (purple). Additional sensitivity analyses examined subgroups of children: (1) meeting the clinical definition of short stature at the initial visit (height SDS ≤  − 2), (2) with a diagnosis of a primary growth disorder, and (3) with a diagnosis of a secondary growth disorder

We then examined whether the PGS_height_ could be used to distinguish between patients with pathology (i.e., primary/secondary growth disorders) and those without pathology who have no other identifiable cause (i.e., ISS-NF). This could suggest that some patients with ISS-NF carry a polygenic form of short stature that is not captured by the MPH. In the analysis subset, there were 201 participants diagnosed with a primary or secondary growth disorder (65.0% of 309) and 108 participants had ISS-NF (35.0%). Polygenic predisposition to short stature (PGS_height_) was associated with a lower likelihood of a diagnosis of a pathologic growth disorder (odds ratio [OR], 0.62 [0.46–0.82]; *p* = 5.5 × 10^−4^) (Fig. 5A). To examine improvement in discrimination, we tested the addition of the PGS_height_ to a baseline model that included MPH. Adding the PGS_height_ improved the AUC from 0.61 to 0.65 (ΔAUC =  + 0.04 [95% CI, 0.01–0.11], *p* = 0.01) (Fig. 5B, Additional file 1: Table S4). In sensitivity analyses, the ΔAUC remained significant when additionally adjusting for a child’s initial visit height. When added to a model with both initial visit height and MPH, the PGS_height_ improved the AUC from 0.64 to 0.68 (+ 0.04 [0.004–0.095]; *p* = 0.02) (Additional file 1: Table S5).

We then separately tested the ability of the PGS_height_ to distinguish between participants with ISS-NF and those diagnosed with either monogenic (primary) or non-monogenic (secondary) growth disorders. Addition of the PGS_height_ was associated with significant improvements in discrimination for both subgroups (Fig. 5, Additional file 1: Table S4). The improvement was strongest for primary growth disorders with the PGS_height_ improving the AUC from 0.67 to 0.74 (+ 0.07 [0.02–0.17]; *p* = 0.003). The improvements in discrimination were also significant in the sensitivity analyses that additionally adjusted for a child’s initial visit height (Fig. S9, Additional file 1: Table S5).

We then calculated a simple, PGS-based height difference measure (PGS-predicted height − initial visit height) that could be used in clinical settings and tested the performance characteristics of this classifier. The optimal threshold for the PGS-based height difference measure was − 2.06 s.d. (median [bootstrapped 95% CI, − 2.42 to − 1.72]). At the optimal threshold, the sensitivity was 58.0% (37.8–77.1%) and the specificity was 59.5% (37.1–82.1%) for identifying patients with any growth disorder. Sensitivity was highest in the height SDS <  − 2 subset (68.0% [50.9–85.2%]), while specificity was highest when distinguishing between patients with ISS-NF and primary growth disorders (81.1% [65.1–91.9%]). We then compared the PGS_height_ performance to the performance of MPH and an average of PGS_height_ and MPH (Additional file 2: Figs. S10 and S11). Across all subgroup analyses, sensitivity was higher when using the PGS_height_ compared to the MPH. Specificity was generally lower when using the PGS_height_.

In summary, the addition of the PGS_height_ improves discrimination between ISS-NF and growth disorders when combined with the standard MPH model. The PGS_height_ is most effective in distinguishing between ISS-NF and primary growth disorders, which are often due to monogenic forms of short stature.

## Discussion

This study evaluates the clinical utility of incorporating PGS_height_ into the diagnostic process for pediatric short stature. When familial origin is defined by mid-parental height (MPH), children with both familial (ISS-F) and non-familial ISS (ISS-NF) had a lower PGS-predicted height than children with other etiologies. Further, children with ISS-NF had a comparable PGS-predicted height to children with ISS-F. The primary diagnostic difference between these groups is that children with ISS-F manifest stature in line with their parents, while children with ISS do not. To further explore this difference, we directly compared height predictions based on the PGS_height_ to those based on mid-parental height. Using mid-parental height, children with ISS were predicted to have heights near the adult population mean. However, children were predicted to be significantly below the mean when using the PGS_height_. To test the clinical implications of this discordance, we examined whether the PGS_height_ could distinguish between children diagnosed with ISS-NF and pathologic growth disorders. The PGS_height_ was able to discriminate the two groups, while MPH could not. This improvement was particularly pronounced for distinguishing between children with primary growth disorders (e.g., monogenic syndrome) and ISS-NF. These findings suggest that mid-parental height estimates may miss genetic factors captured by the PGS_height_, thereby leading to a misclassified ISS-NF diagnosis. Incorporating polygenic height predictors could improve diagnostic accuracy, ultimately improving clinical outcomes for children with ISS-NF.

Studies in prospective, population-based cohorts have shown that polygenic scores for height can improve adult height predictions and identify children at risk for short stature in adulthood [14, 15]. Our study extends this work to children with short stature who are undergoing a diagnostic evaluation seeking an explanation for their height. Short stature comprises a heterogeneous collection of diagnoses, ranging from benign conditions such as ISS-F to underlying diseases [9, 34, 35]. Our studies assess the feasibility of using a PGS_height_ to identify patients at lower risk for growth disorders.

An adult height estimate based on parental heights (MPH) is typically used to estimate a child’s genetically determined adult height potential. This study highlights the potential limitations of MPH and suggests that it may not accurately measure polygenic drivers of growth for all children presenting for evaluation. This discordance may be particularly apparent among children referred for an endocrine evaluation as one indication for referral is discordance between the child’s height and their MPH. Consistently, children with ISS-NF in this cohort had average parental heights, so short stature was not expected by the clinician based on MPH alone.

The inherent uncertainty of an ISS-NF diagnosis can often lead to prolonged monitoring for latent disease that has not revealed itself [36]. In this study, we show that the PGS_height_, but not the MPH, improved discrimination of children with underlying disease from those children that remain with ISS even after a specialist’s evaluation. While significant, the magnitude of the improvements was relatively modest. The most likely explanation for the modest improvement is that ISS represents a heterogeneous collection of etiologies including undiagnosed rare genetic variation [11]. An unmeasured polygenic predisposition could be considered an additional, testable cause of short stature in this population.

Discordance between parental and polygenic height prediction could have many explanations including misestimation of parental heights due to inaccurate reporting or misattributed paternity [37]. In one study, inaccurate self-reporting of parental heights was common, with 30% of couples having a self-reported MPH that was more than 2 cm different than measured MPH [38, 39]. In these instances, the PGS_height_ could be a valuable addition to a diagnostic algorithm, in particular for those children diagnosed with ISS-NF. Another application would be for adopted children with unknown parental heights, for whom MPH estimates are not available.

Recessive genetic variation or de novo genetic mutations are well recognized but often underdiagnosed causes of ISS. A recent meta-analysis has shown that exome sequencing and chromosomal microarray can identify a genetic etiology in 27.1% and 13.6% of patients, respectively, that were previously diagnosed with ISS [11]. We show that children diagnosed with either ISS-F or ISS-NF at higher monogenic risk, defined as having a short parent, have a lower polygenic predisposition than children without a short parent. This suggests that the PGS_height_ could be used with parental heights to identify children with ISS at higher risk of monogenic short stature. For instance, a child who has a parent with short stature but does not have a polygenic predisposition to short stature is more likely to benefit from sequencing than a child with a polygenic predisposition with normal stature parents [40]. On the other hand, the PGS_height_ could be used to re-classify some children with ISS-NF that have a particularly strong polygenic predisposition. A diagnosis of “polygenic short stature,” while yet undefined, could provide reassurance to clinicians and parents that a patient’s short stature is of a benign etiology [13, 41].

In this EHR-based cohort, we provide further evidence that polygenic short stature is a significant cause of ISS-NF as hypothesized in previous studies [14]. However, future research is needed before implementation of the PGS_height_ in this clinical setting to identify cases of polygenic short stature. Our study utilizes the final diagnosis of the endocrinologist as the primary outcome, but we are limited by the incomplete ascertainment of underlying pathologic causes of disease. Longitudinal, prospective studies are needed to more fully quantify the accuracy of the PGS_height_ for identification of cases with eventual manifestation of growth disorders in adulthood. Parental height data were extracted from real-world data entered in the EHR by the clinician, so assessment of parental heights was not standardized which could lead to inaccurate estimation of the MPH. We were only able to assess monogenic risk superficially using the presence of a parent with short stature as a proxy as suggested by the American College of Medical Genetics [12]. However, whole genome sequencing is needed for complete ascertainment of monogenic short stature. We were unable to assess the clinical performance of the PGS_height_ in participants of other ancestry groups as there were fewer than 10 cases of ISS-NF in each group. More diverse cohort studies will be needed to assess the portability of using the PGS_height_ to distinguish between patients with polygenic short stature and short stature due to growth disorders.

## Conclusions

In summary, we show that the PGS_height_ measures a polygenic predisposition to shorter height in children with non-familial ISS (ISS-NF) that is not captured fully by MPH. Addition of the PGS_height_ improves discrimination of children with ISS-NF from children with short stature due to pathology. Incorporating the PGS_height_ into the clinical evaluation of ISS could aid in the identification of polygenic short stature that is not recognized when using the traditional measure of predisposition, MPH. This could prove valuable for children and clinicians alike by helping to clarify the benign nature of a child’s short stature, potentially allowing for the de-escalation of unnecessary diagnostic testing.

## Supplementary Information

Additional file 1: Supplementary Tables. This file contains all Supplementary Tables and their corresponding legends.

Additional file 2: Supplementary Methods and Figures. This file contains all Supplementary Figures, their corresponding legends, and Supplementary Methods describing height standardization and polygenic height predictions.

## Data Availability

Subject-level access to BioVU clinical and genetic data is controlled by the BioVU data repository (https://victr.vumc.org/biovu-description/). Datasets of participant-level phenotype data to replicate the primary findings presented here will be made available upon request from the repository (biovu@vumc.org). Institutional IRB approval, data use agreements, and administrative and scientific reviews are required prior to using individual-level data from BioVU.
