# Supplementary material for: A polygenic score for height identifies an unmeasured genetic predisposition among pediatric patients with idiopathic short stature
Source: Genome Med. 2025 Mar 19;17:23. doi: 10.1186/s13073-025-01455-3 (PMC11924680; doi:10.1186/s13073-025-01455-3)

SUPPLEMENTAL CONTENT

Supplemental Methods

Figure S1: Initial visit height by short stature etiology

Figure S2: Subset with initial visit height SDS < -2: PGS_height_ by short stature etiology

Figure S3: Subset with initial visit height SDS < -2: Discrepancy between predicted heights generated by PGS_height_ and MPH

Figure S4: Discrepancy between predicted heights generated by PGS_height_ and MPH among children with primary or secondary growth disorders, by etiology

Figure S5: Subset with initial visit height SDS < -2: Discrepancy between predicted heights generated by PGS_height_ and MPH among children with primary or secondary growth disorders, by etiology

Figure S6: Subset with adult heights: Discrepancy between measured and predicted adult heights generated by PGS_height_ and MPH.

Figure S7: Subset with adult heights: Accuracy of MPH and PGS_height_ for prediction of adult height among patients with ISS-F or ISS-NF

Figure S8: Subset with ISS-F or ISS-NF: Comparison of height predictions by risk for monogenic short stature, defined as having a parent with height SDS < -2

Figure S9: Model discrimination between ISS-NF and pathologic growth disorders with addition of initial visit height, MPH and PGS_height_

Figure S10: Comparing optimal thresholds (A) and model discrimination (B) of MPH- and PGS-based height differences for distinguishing between ISS-NF and pathologic growth disorders

Figure S11: Comparing performance metrics (PPV, NPV, sensitivity, specificity) of MPH- and PGS-based height difference to distinguish between ISS-NF and pathologic growth disorders

Supplemental Tables (.xlsx file)

Table S1: Phecodes related to pathologic causes of short stature used to exclude subjects from the overall population of adults and children

Table S2: Phecodes related to pathologic causes of short stature used to exclude subjects from the overall population of adults and children

Table S3: Descriptive statistics for general population cohort

Table S4: Results from discrimination analyses in the primary analysis testing the benefit of adding PGS_height_ to MPH

Table S5: Results from discrimination analysis in the sensitivity analysis which additionally adjusted the baseline model for initial visit height

Supplemental Methods

Standardizing adult height data to a common reference standard: Age- and sex- standardized pediatric height measurements are typically based upon the Centers for Disease Control and Prevention (CDC) reference standards for the U.S. population. To standardize adult height measurements, a reference population was derived from National Health and Nutrition Examination Survey (NHANES) between 2001 and March 2020. Sex-specific means and standard deviations of measured height were calculated for the NHANES population, accounting for sample weights provided by NHANES and complex survey design, as instructed.^21^ Adult heights were converted to centimeters and an SDS was calculated by subtracting adult heights by the respective sex-specific NHANES mean height and dividing this difference by the sex-specific NHANES standard deviation.

Predicting expected adult height using a polygenic score for height: Mid-parental height (MPH) estimates reflect a child’s predicted final height, whereas a standardized polygenic score (PGS_height_), reflects a child’s expected deviation from the adjusted average height of participants in the height GWAS. To convert the PGS_height_ estimate to an expected adult height, a linear prediction model for height SDS was developed among BioVU adults of European ancestry (N=33,637). For each adult, height SDS was calculated as the median across all measurements. A multivariable linear regression model was used, regressing a person’s median height SDS on their PGS_height_ with the top 10 within-ancestry principal components (PCs) as covariates. For every 1 standard deviation (s.d.) in the PGS_height_, there was a 0.63 s.d. increase in height. The model explained 39.1% of the variance in height in this population, with the PGS_height_ accounting for the majority of this variance (Adjusted R^2^=38.1%). This model was then used to compute an expected adult height SDS for each child in the short stature cohort.

Figure S1: Initial visit height by short stature etiology. Violin plots comparing initial visit height SDS by short stature etiology in the short stature cohort. The overall pediatric population is included as a reference. The clinical threshold for short stature (≥2 s.d. below the CDC reference standard mean) is labelled with a red, dashed line.


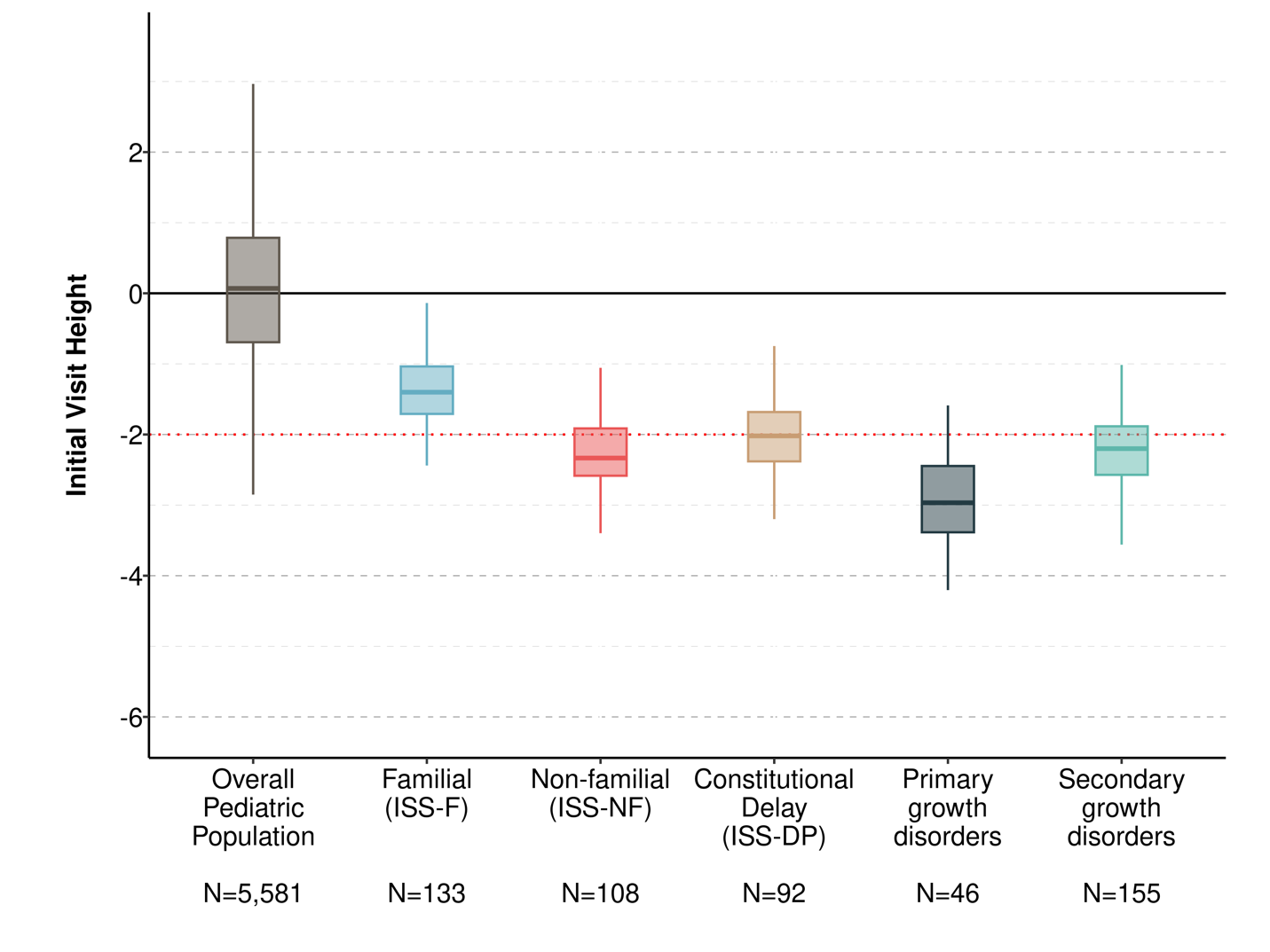


Figure S2: Subset with initial visit height SDS < -2: PGS_height_ by short stature etiology. Violin plots comparing PGS_height_ SDS by short stature etiology in the short stature cohort. The overall pediatric population is included as a reference.


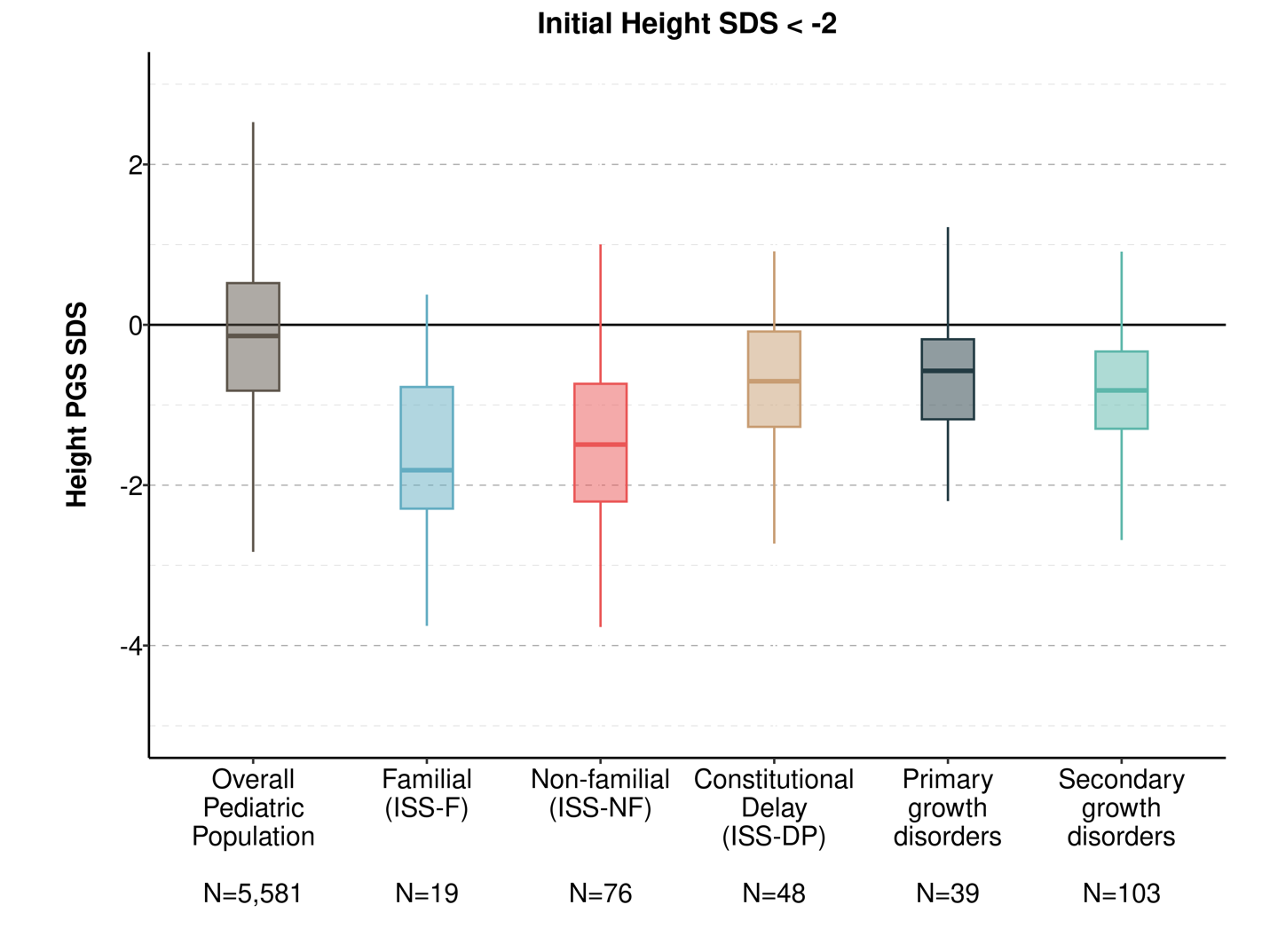


Figure S3: Subset with initial visit height SDS < -2: Discrepancy between predicted heights generated by PGS_height_ and MPH. Violin plots comparing height predictions by method (MPH and PGS_height_) for each sub-group based on short stature etiology. The PGS_height_ was converted to a predicted adult height by modelling height as a function of PGS_height_ and 10 principal components in BioVU adults. The median difference in height predictions using the PGS_height_ and MPH is shown above each subgroup. Paired samples Wilcoxon rank sum tests were used to test the significance of within-person differences. ***: Wilcoxon p<0.001.


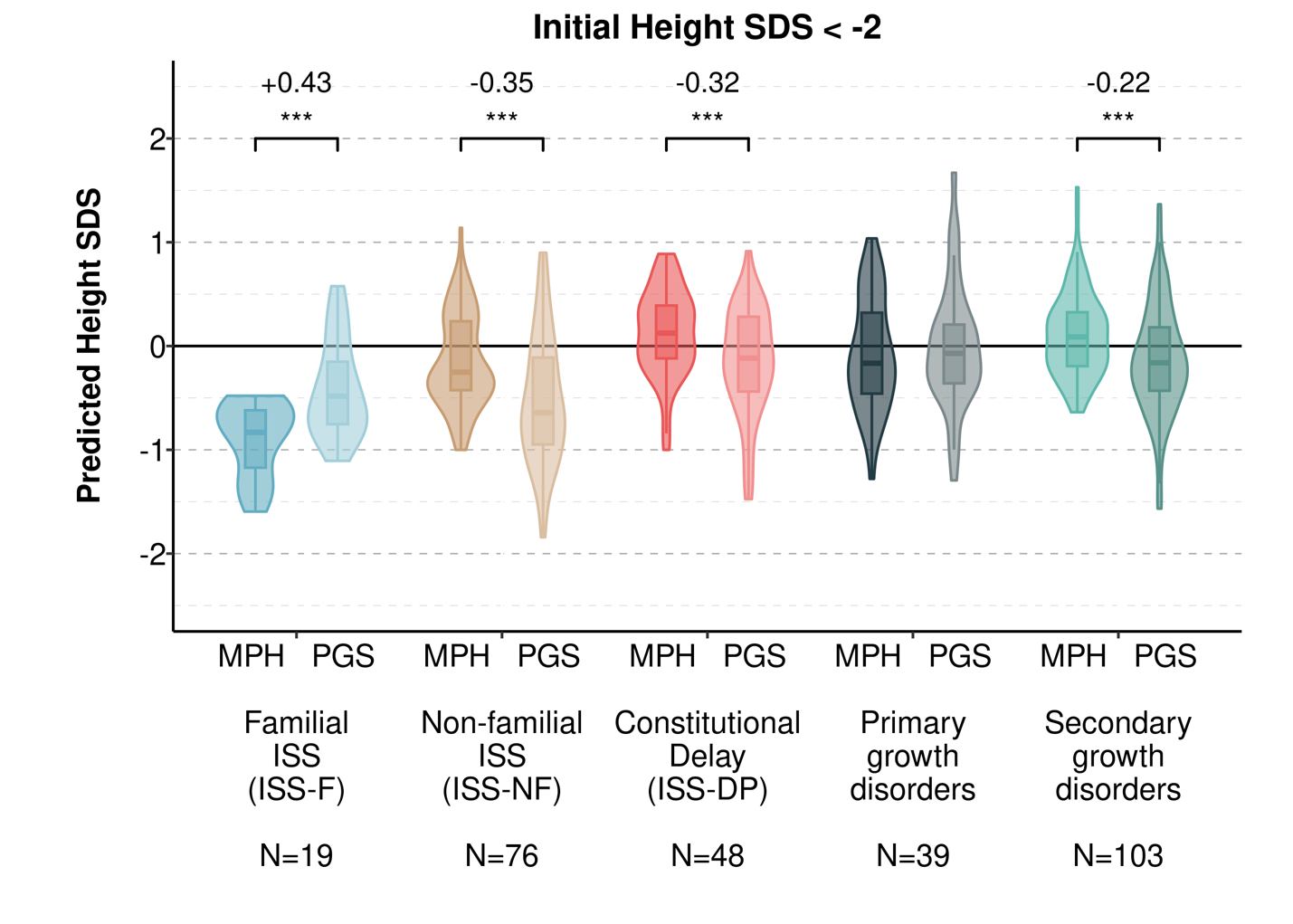


Figure S4: Discrepancy between predicted heights generated by PGS_height_ and MPH among children with primary or secondary growth disorders, by etiology. Violin plots comparing height predictions by method (MPH and PGS_height_) for each growth disorder. Growth hormone deficiency excludes participants diagnosed with growth hormone deficiency clinically with a normal (≥10ng/ml) peak GH response to provocation. The PGS_height_ was converted to a predicted adult height by deriving a model predicting height as a function of PGS_height_ and 10 principal components in the population of BioVU adults. The median difference in height predictions using the PGS_height_ and MPH is shown above each subgroup. Paired samples Wilcoxon rank sum tests were used to test the significance of within-person differences. ***: Wilcoxon p<0.001.


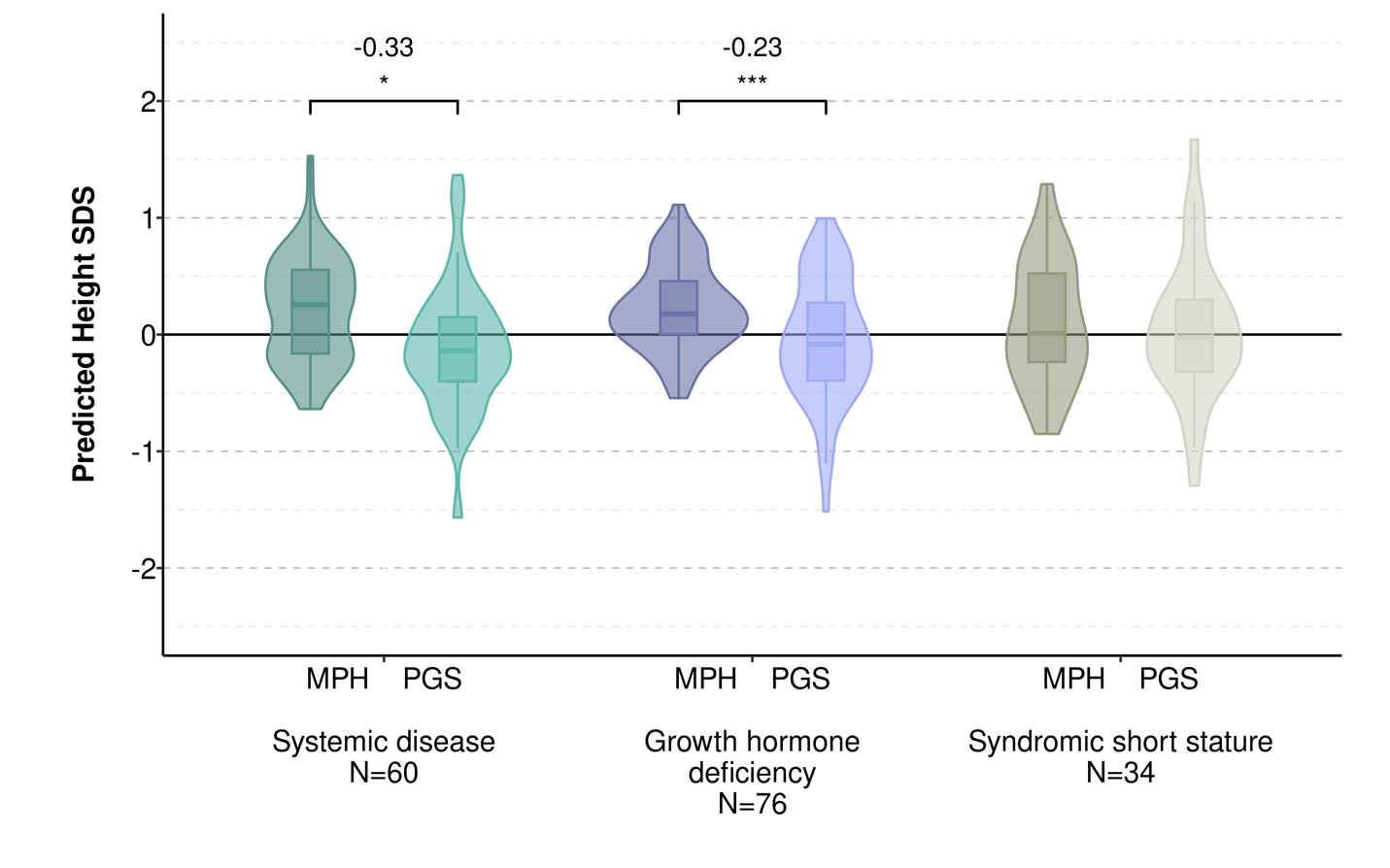


Figure S5: Subset with initial visit height SDS < -2: Discrepancy between predicted heights generated by PGS_height_ and MPH among children with primary or secondary growth disorders, by etiology. Violin plots comparing height predictions by method (MPH and PGS_height_) for each growth disorder. Growth hormone deficiency excludes participants diagnosed with growth hormone deficiency clinically with a normal (≥10ng/ml) peak GH response to provocation. The PGS_height_ was converted to a predicted adult height by deriving a model predicting height as a function of PGS_height_ and 10 principal components in the population of BioVU adults. The median difference in height predictions using the PGS_height_ and MPH is shown above each subgroup. Paired samples Wilcoxon rank sum tests were used to test the significance of within-person differences. ***: Wilcoxon p<0.001.


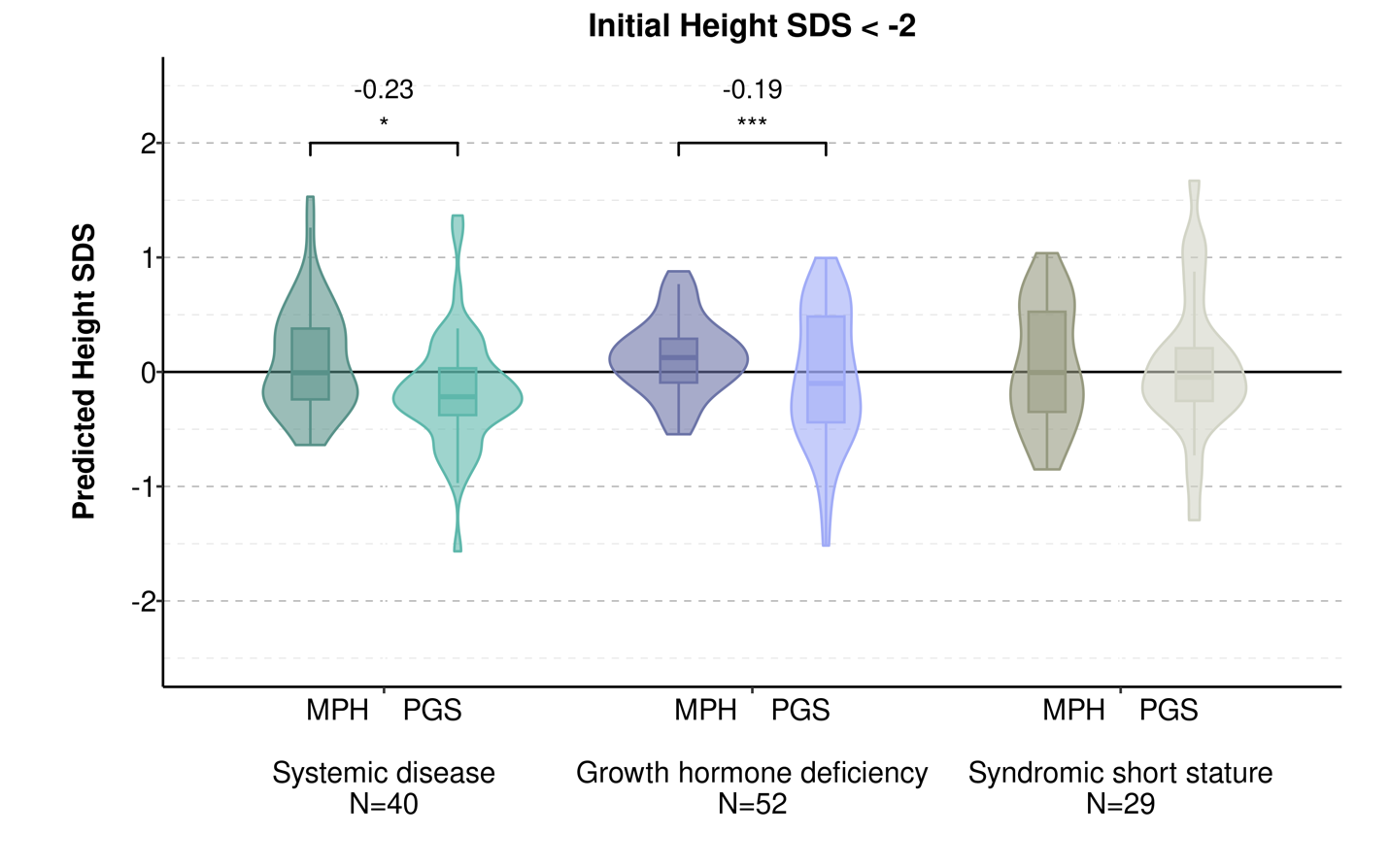


Figure S6: Subset with adult heights: Discrepancy between measured and predicted adult heights generated by PGS_height_ and MPH. A) Scatter plot showing the relationship between predicted heights using MPH and PGS_height_ in the 534 participants in the short stature cohort. B) Box plots comparing the difference between actual and predicted adult heights when using the MPH and PGS_height_ in a subset of 122 participants with adult height measurements available.


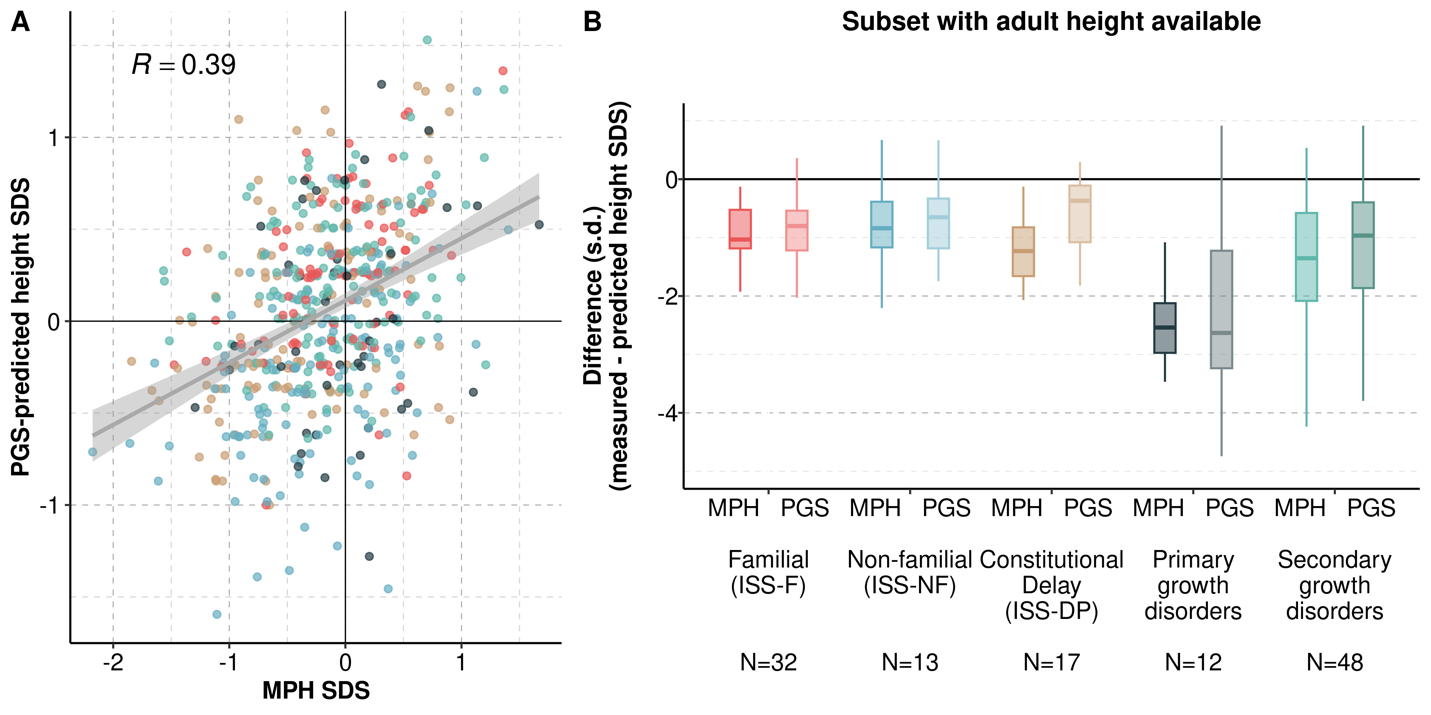


Figure S7: Subset with adult heights: Accuracy of MPH and PGS_height_ for prediction of adult height among patients with ISS-F or ISS-NF. Plots illustrate bootstrapped R^2^ estimates of models using MPH and PGS_height_ as linear predictors of a person’s measured adult height. An integrated model using both predictors was also tested. Bootstrapping with 5,000 replicates was performed to calculate the median and 95% confidence interval of the R^2^ for each model.


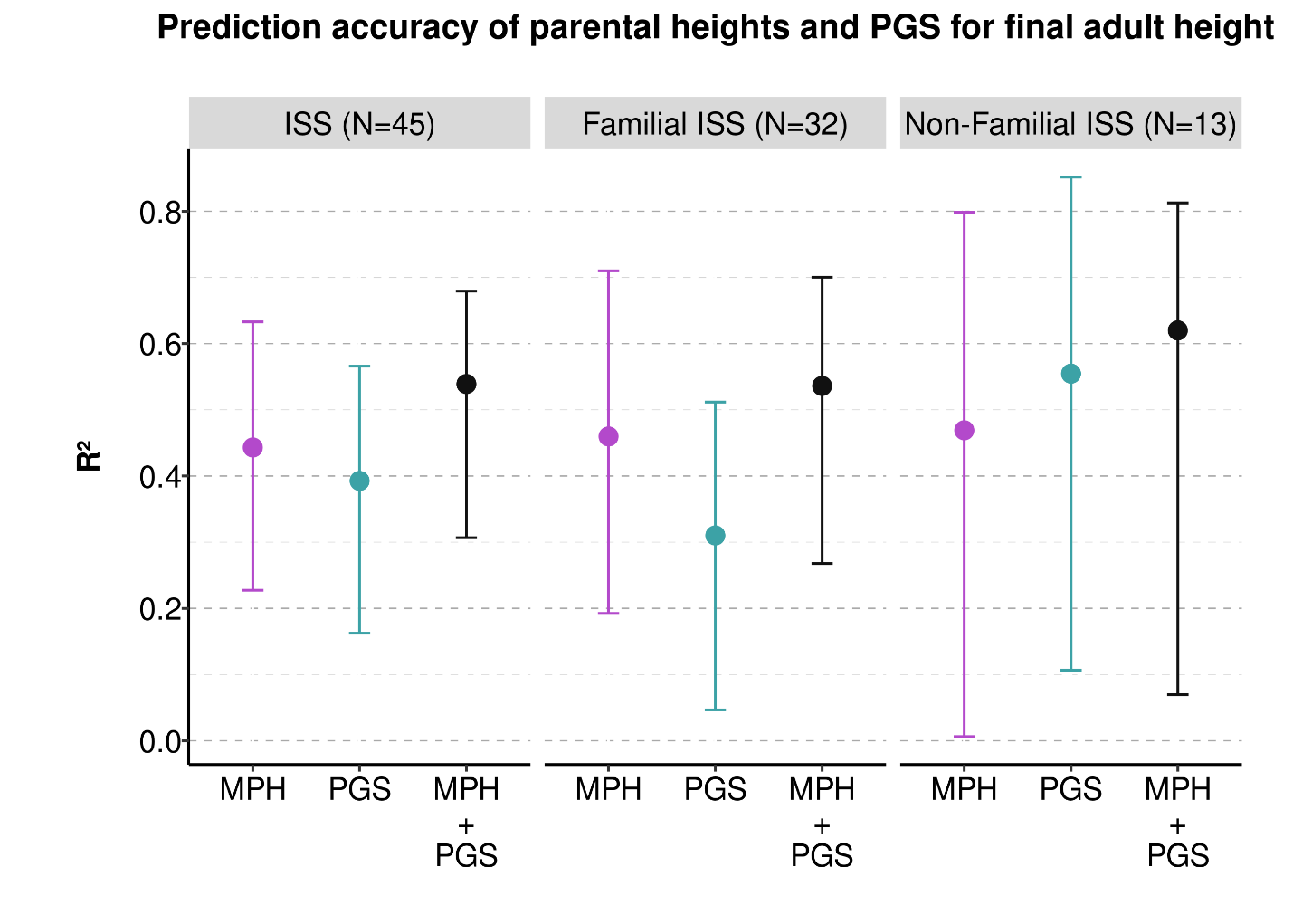


Figure S8: Subset with ISS-F or ISS-NF: Comparison of height predictions by risk for monogenic short stature, defined as having a parent with height SDS < -2. Box plots comparing height predictions by method (MPH and PGS_height_) for each sub-group based on whether the participant has a height SDS < -2. The PGS_height_ was converted to a predicted adult height by modelling height as a function of PGS_height_ and 10 principal components in BioVU adults. Paired samples Wilcoxon signed rank tests were used to test the significance of within-person differences. Wilcoxon rank sum tests were used to test the significance of PGS_height_ differences between the groups. ***: Wilcoxon p<0.001.


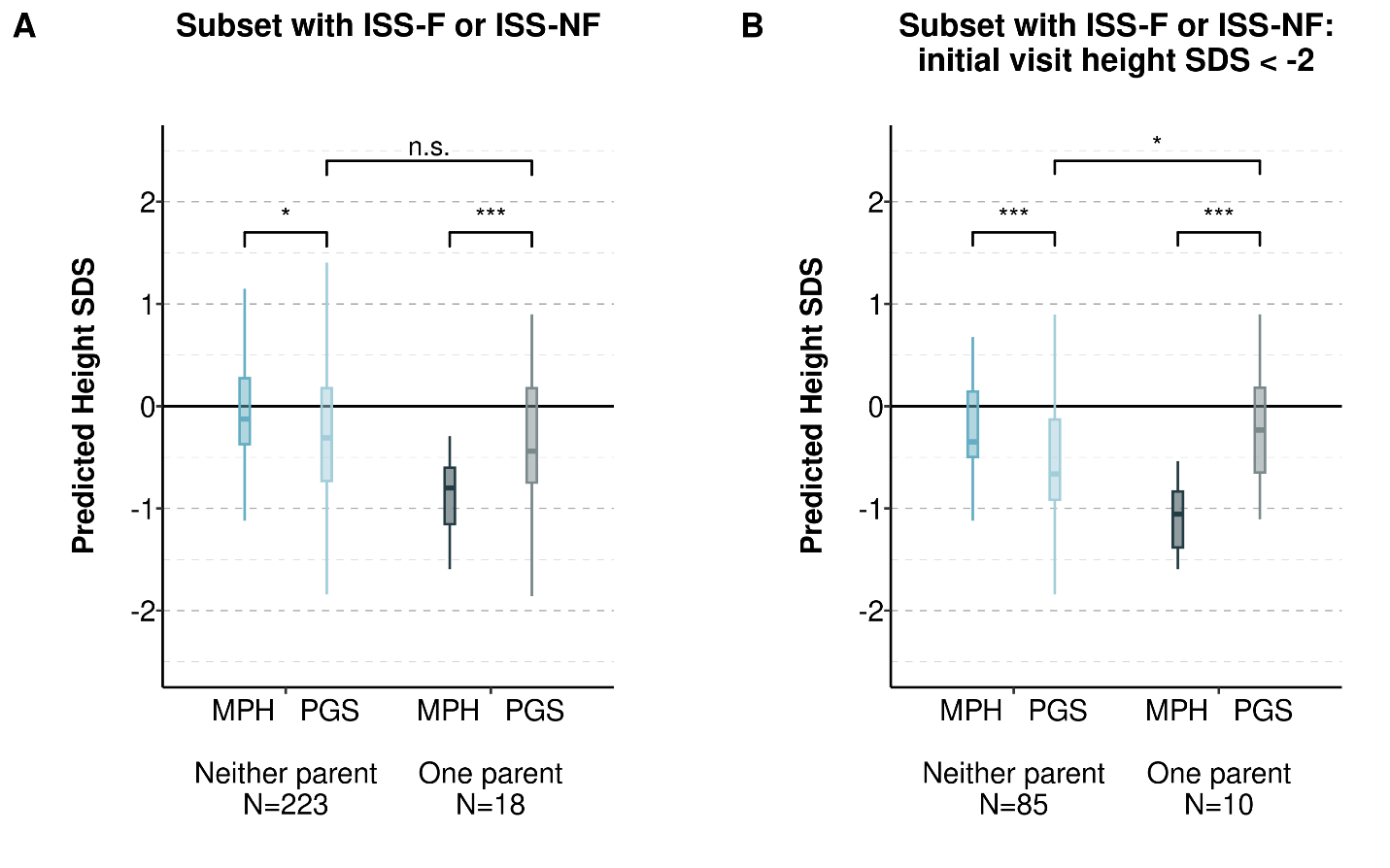


Figure S9: Model discrimination between idiopathic and pathologic growth disorders with addition of initial visit height, MPH and PGS_height_. Forest plot comparing discrimination (AUC) between ISS-NF and pathologic growth disorders when using a model with only age, sex, and 10 PCs (black), with addition of the initial visit height (yellow), with addition of the MPH (blue), and with addition of the PGS_height_ (purple). Additional sensitivity analyses examined subgroups of children: 1) meeting the clinical definition of short stature at the initial visit (height SDS ≤ -2), 2) with a diagnosis of a primary growth disorder, and 3) with a diagnosis of a secondary growth disorder.


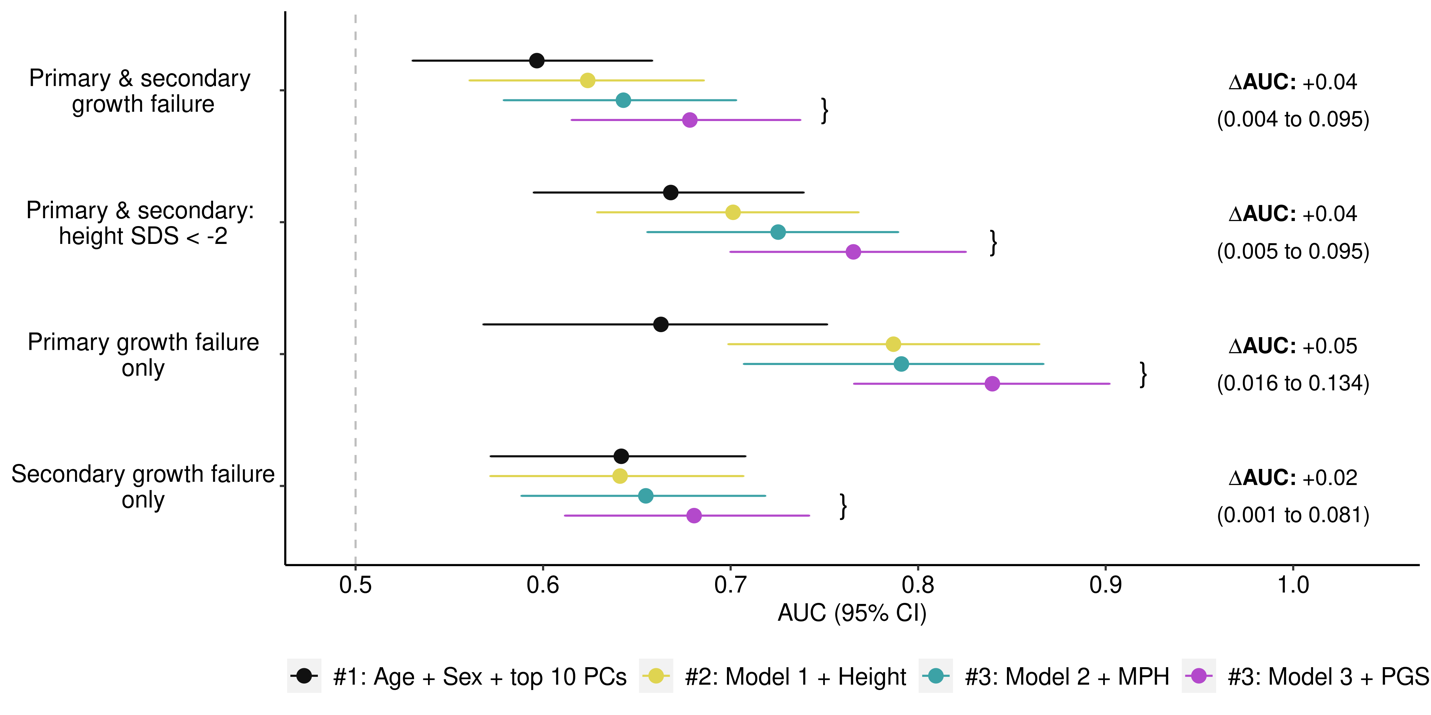


Figure S10: Comparing optimal thresholds (A) and model discrimination (B) of MPH- and PGS-based height differences for distinguishing between idiopathic and pathologic growth disorders. Plots showing optimal thresholds of PGS- and MPH-based height differences for discriminating between ISS-NF and pathologic growth disorders. The average of PGS- and MPH-based height differences was also evaluated. Optimal thresholds were estimated using the Youden index and bootstrapping was performed using 5,000 replicates to estimate the distribution. Additional sensitivity analyses examined subgroups of children: 1) meeting the clinical definition of short stature at the initial visit (height SDS ≤ -2), 2) with a diagnosis of a primary growth disorder, and 3) with a diagnosis of a secondary growth disorder.


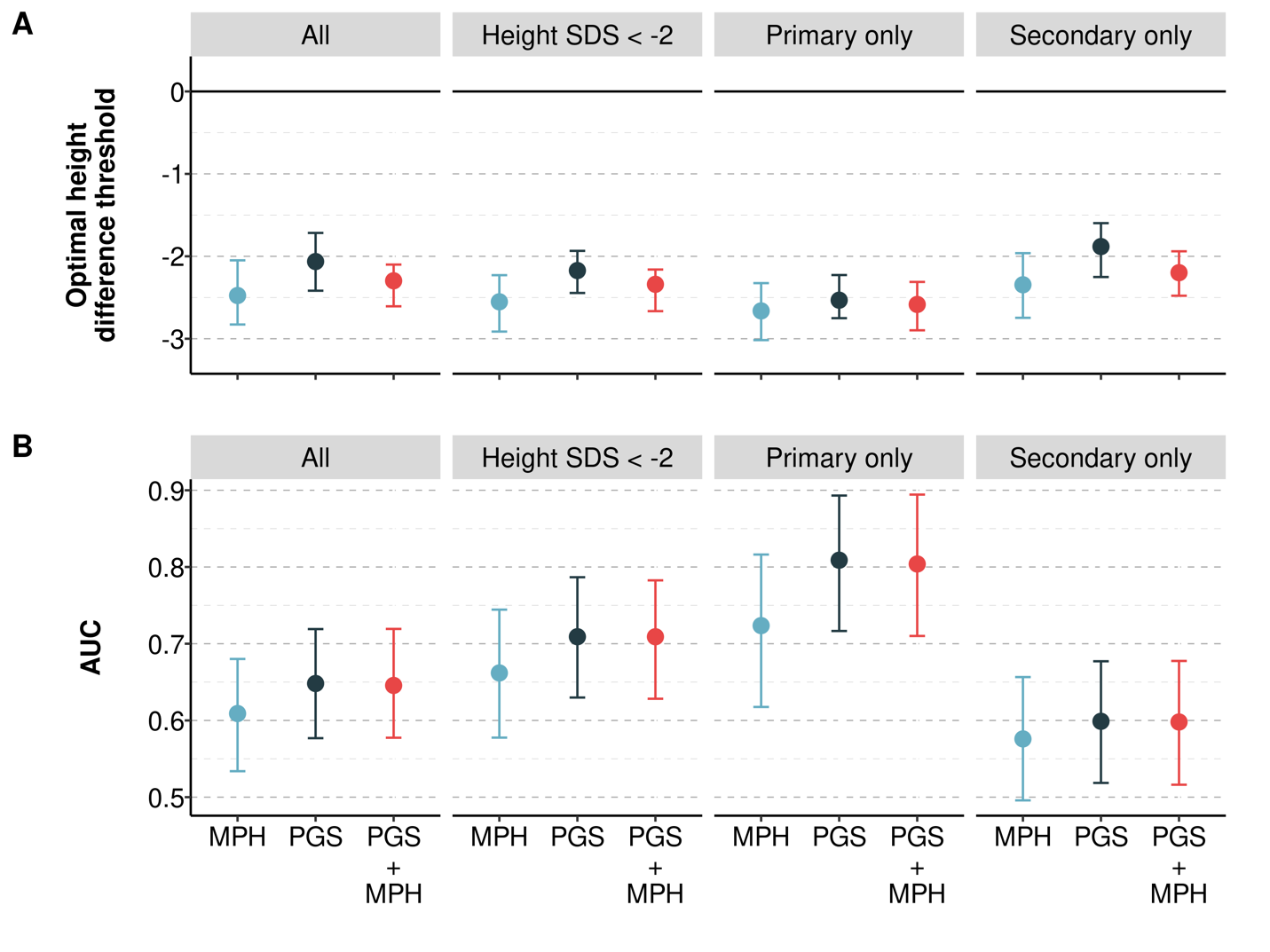


Figure S11: Comparing performance metrics of MPH- and PGS-based height difference to distinguish between ISS-NF and pathologic growth disorders. Plots showing A) PPV, B) NPV, C) sensitivity, and D) specificity of PGS-based height difference, MPH-based height difference, and the average of the two for discriminating between ISS-NF and pathologic growth disorders. Bootstrapping was performed with 5,000 replicates to estimate the optimal threshold and the performance characteristics. Additional sensitivity analyses examined subgroups of children: 1) meeting the clinical definition of short stature at the initial visit (height SDS ≤ -2), 2) with a diagnosis of a primary growth disorder, and 3) with a diagnosis of a secondary growth disorder.


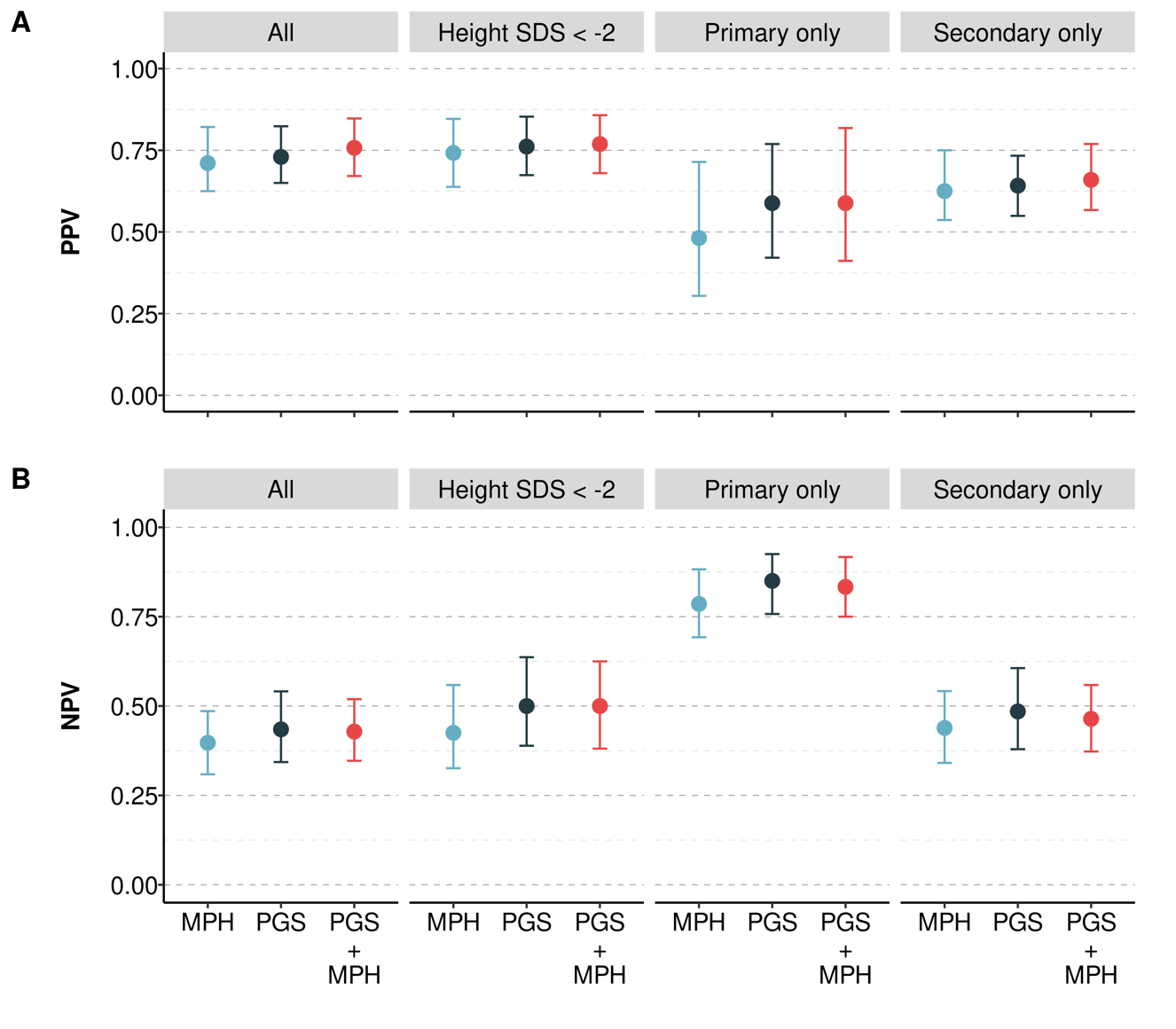


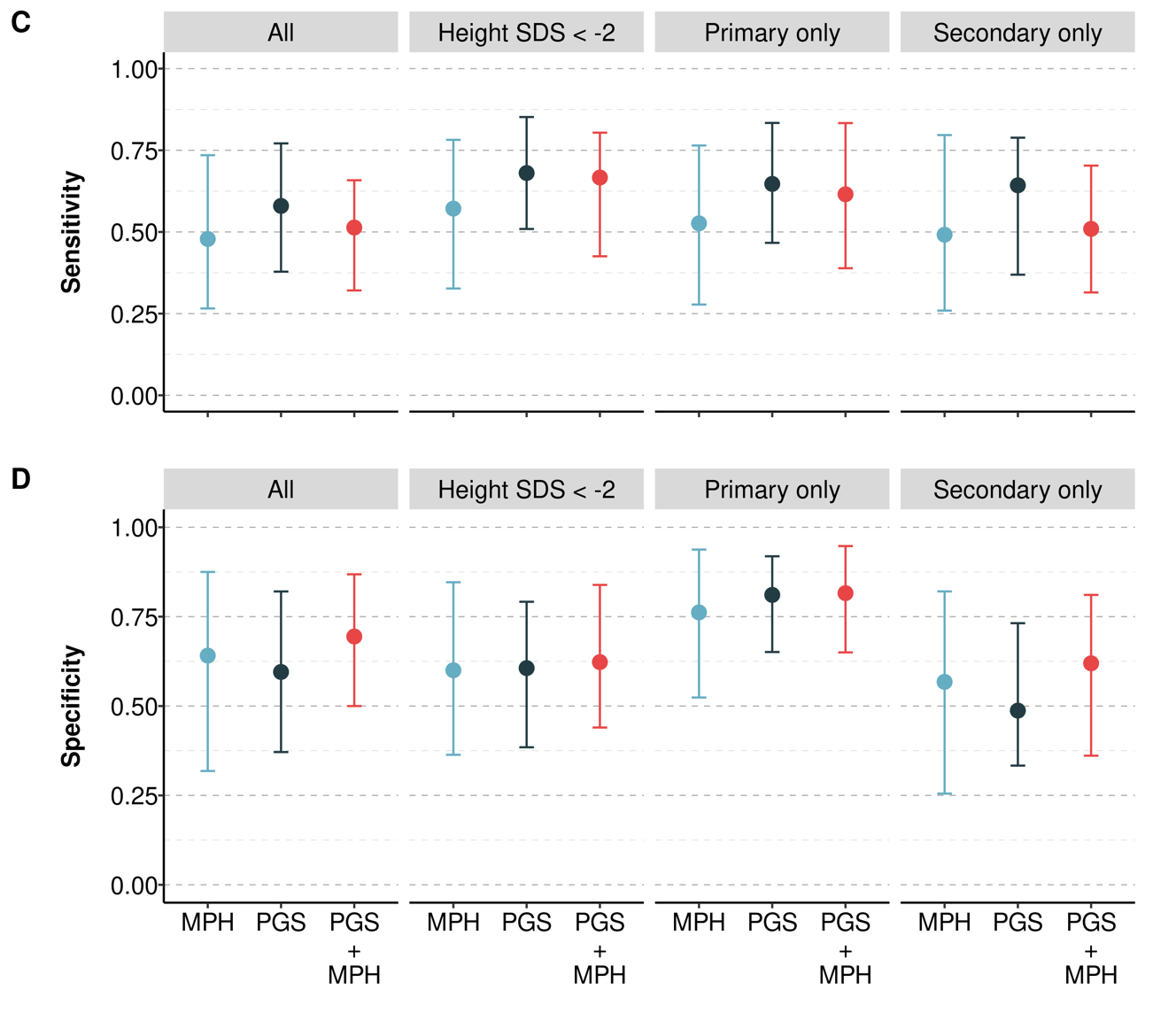

Supplement: Supplementary file 2 — Additional file 2: Supplementary Methods and Figures. This file contains all Supplementary Figures, their corresponding legends, and Supplementary Methods describing height standardization and polygenic height predictions. [file 13073_2025_1455_MOESM2_ESM.docx]
